# Metabolic engineering in *Streptomyces albidoflavus* for the biosynthesis of the methylated flavonoids sakuranetin, acacetin, and genkwanin

**DOI:** 10.1186/s12934-023-02247-3

**Published:** 2023-11-14

**Authors:** Álvaro Pérez-Valero, Suhui Ye, Patricia Magadán-Corpas, Claudio J. Villar, Felipe Lombó

**Affiliations:** 1 Research Group BIONUC (Biotechnology of Nutraceuticals and Bioactive Compounds), Departamento de Biología Funcional, Área de Microbiología, Universidad de Oviedo, Oviedo, Principality of Asturias Spain; 2 IUOPA (Instituto Universitario de Oncología del Principado de Asturias), Oviedo, Principality of Asturias Spain; 3 ISPA (Instituto de Investigación Sanitaria del Principado de Asturias), Oviedo, Principality of Asturias Spain

**Keywords:** Flavonoid, Genome editing, Biosynthesis, Methyltransferase, Co-culture

## Abstract

Flavonoids are important plant secondary metabolites showing antioxidant, antitumor, anti-inflammatory, and antiviral activities, among others. Methylated flavonoids are particularly interesting compared to non-methylated ones due to their greater stability and intestinal absorption, which improves their oral bioavailability. In this work we have stablished a metabolic engineered strain of *Streptomyces albidoflavus* with enhanced capabilities for flavonoid production, achieving a 1.6-fold increase in the biosynthesis of naringenin with respect to the parental strain. This improved strain, *S. albidoflavus* UO-FLAV-004, has been used for the heterologous biosynthesis of the methylated flavonoids sakuranetin, acacetin and genkwanin. The achieved titers of sakuranetin and acacetin were 8.2 mg/L and 5.8 mg/L, respectively. The genkwanin titers were 0.8 mg/L, with a bottleneck identified in this producing strain. After applying a co-culture strategy, genkwanin production titers reached 3.5 mg/L, which represents a 4.4-fold increase. To our knowledge, this study presents the first biosynthesis of methylated flavonoids in not only any *Streptomyces* species*,* but also in any Gram-positive bacteria.

**Supplementary Information:**

The online version contains supplementary material available at 10.1186/s12934-023-02247-3.

## Background

**Fig. 1:**
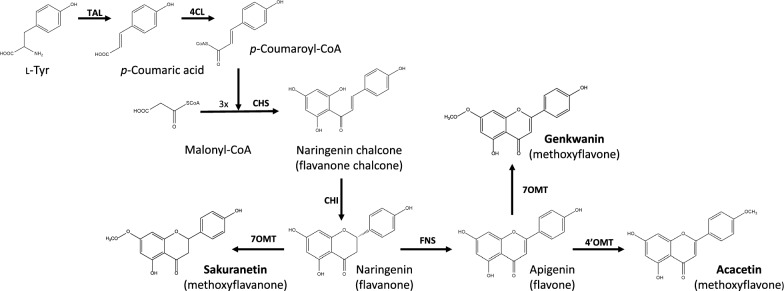
Biosynthetic pathway for the heterologous biosynthesis of sakuranetin, acacetin and genkwanin. Tyrosine ammonia-lyase (TAL); 4-Coumaroyl-CoA ligase (4CL); Chalcone synthase (CHS); Chalcone isomerase (CHI); Flavone synthase (FNS); 4’-O-methyltransferase (4’OMT); 7-O-methyltransferase (7OMT)

Flavonoids are a large family of nutraceuticals widely distributed in plant cells, including dietary plants [1–5]. *In planta*, flavonoids are synthesized by complexes of various enzymes that are present on the cytosolic face of the endoplasmic reticulum membranes. The first steps in flavonoid biosynthesis are part of the phenylpropanoid pathway, which converts L-phenylalanine into 4-coumaroyl-CoA, via three enzymatic steps [6, 7]. These first three enzymatic steps are catalyzed by phenylalanine ammonia lyase (PAL), cinnamate 4-hydroxylase (4CH) and 4-coumaroyl-CoA ligase (4CL) (Fig. 1). However, in bacteria, the use of tyrosine ammonia lyase (TAL) is preferred for heterologous biosynthesis, instead of PAL, as starting from L-Tyr removes the need for the 4CH activity since this amino acid is already hydroxylated at the required position [8, 9]. In the next enzymatic step, chalcone synthase (CHS) condenses a molecule of 4-cumaroyl-CoA with three molecules of malonyl-CoA, generating naringenin chalcone, the basic carbon skeleton for more than 9.000 known flavonoids in nature [2, 6, 7, 10]. The heterocycle closure in naringenin chalcone is catalyzed by a chalcone isomerase (CHI), generating naringenin, a universal flavanone precursor.

Flavonoids have been investigated as antitumor [11–13], antimicrobial, antiangiogenic [12, 13], antioxidant and neuroprotective compounds, among many other bioactivities [14]. Methylated flavonoids, which also possess these interesting properties [15], are more stable, show improved oral bioavailability, better absorption, and enhanced membrane transport [16]. The two types of methylation patterns in flavonoids are *C-*methylation and *O-*methylation, in which the methyl moieties are donated by S-adenosylmethionine (SAM) [17]. Despite methylated flavonoids being common in plants, they are less abundant than flavonoid glycosides, which makes them attractive candidates for heterologous production via synthetic biology. In this work, we focus our efforts on the heterologous biosynthesis of the *O-*methylated flavonoids sakuranetin, acacetin and genkwanin, using the microbial factory *Streptomyces albidoflavus*, formerly known as *Streptomyces albus*.

Sakuranetin is a 7-*O-*methyl flavanone derived from naringenin (Fig. 1). Sakuranetin is found in different species of the *Prunus* genus, as well as in *Baccharis retusa*, *Ribes nigrum* [18] and *Oryza sativa* [19]. It has been shown to have different bioactivities, such as anti-inflammatory [20], antidiabetic [21], antiviral [22, 23] or antifungal [24, 25].

Acacetin has been isolated from *Chrysanthemum indicum*, safflower, *Calamintha* and *Linaria* species. It is a 4’-*O-*methyl flavone derived from apigenin (Fig. 1), and it shows anti-cancer activity [26–30], as well as neuroprotective effects, making it a potential therapeutic agent for neurodegenerative diseases like Alzheimer’s and Parkinson’s [22, 31]. In addition, acacetin possesses therapeutic potentials in rescuing neuronal injuries caused by ischemia [32, 33] and it is a potential antidiabetic compound [34].

Genkwanin is a 7-*O*-methylated derivative of the flavone apigenin (Fig. 1), found in *Daphne genkwa*, *Rosmarinus officinalis* and *Cistus laurifolius*. In different pharmacological studies, this compound showed antibacterial, antiplasmodial, radical scavenging, and chemopreventive activities, among others [35].

Different microbial hosts have been used for the biosynthesis of methylated flavonoids, such as *Escherichia coli* [36] and *Saccharomyces cerevisiae* [37]*.* However, to our knowledge, the biosynthesis of methylated flavonoids in Gram-positive bacteria has not been reported, and the use of these bacterial factories can facilitate the further industrialization of these important bioactive compounds [38]. Actinomycetes, such as *S. albidoflavus,* are suitable for genetic engineering and metabolic optimization at different levels such as precursor cytoplasmic pools, export of final products to culture medium, biomass modulation, etc. Furthermore, they constitute the main microbial producers of diverse pharmaceutical drugs and bioactive compounds such as antitumorals, macrolides, aminoglycosides, etc. [39]. Furthermore, the biosynthesis of the central flavonoid naringenin has been observed in *Streptomyces* [40].

The major challenge in the heterologous biosynthesis of flavonoids through synthetic biology is the low production titers, directly associated with the limited availability of intracellular precursors and cofactors. To deal with this problem, different approaches have been adopted to increase flavonoid precursor supplies, such as redirecting central carbon metabolic pathways towards the production of malonyl-CoA [41]; or in the case of L-tyrosine, by removing the feedback inhibition shown by the enzymes DAHP synthase and chorismate mutase in the shikimate pathway [42].

In this work, we have developed different strains of *Streptomyces albidoflavus* using CRISPR-Cas9 technology, in which we deleted endogenous biosynthetic gene clusters (BGCs) in this bacterium than can compete for the two mentioned flavonoid precursors. The new engineered strains have been tested for the biosynthesis of the central flavonoid naringenin, and the best strain has been then selected for the heterologous biosynthesis of sakuranetin, acacetin and genkwanin. In addition, a co-cultivation strategy has been carried out to solve a bottleneck detected during the heterologous biosynthesis of genkwanin. This type of strategy has been used previously to split complex metabolic pathways between different strains or different microbial hosts [43].

## Results

### Deletion of endogenous gene clusters

With the aim of increasing the final flavonoid titers, new strains of *S. albidoflavus* was generated via metabolic engineering. A first genomic modification was performed to generate *S. albidoflavus* UO-FLAV-003 on the previously published parental *S. albidoflavus* UO-FLAV-002 strain [45]. A chromosomal fragment comprising the BGC number 2 (BGC2) predicted by AntiSmash was removed, which encodes polycyclic tetramate macrolactams (PTMs), a type of secondary metabolite showing antibacterial activity [44]. This chromosomal deletion comprised the region from 239,275 to 254,893 bp. This BGC uses as a precursor malonyl-CoA, which is a building block necessary for flavonoid biosynthesis. To check if the deletion of this BGC was useful for enhancing flavonoid biosynthesis, the naringenin BGC was integrated into the *S. albidoflavus* UO-FLAV-003 strain at the chromosomal φC31 attB site, generating the strain *S. albidoflavus* UO-FLAV-003-NAR [45].

The next genomic modification was performed over the *S. albidoflavus* UO-FLAV-003 strain. In this case, the chromosomal fragment comprising BGC number 5 (BGC5) predicted by AntiSmash, encoding the paulomycin biosynthetic pathway, was deleted to generate the strain *S. albidoflavus* UO-FLAV-004. This chromosomal deletion comprised the region from 674,514 to 720,094 bp. The paulomycin BGC uses chorismate as a precursor molecule [46], therefore competing with the biosynthesis of L-tyrosine, a building block necessary for heterologous naringenin biosynthesis. In addition, this BGC also consumes acetyl-CoA, a precursor of malonyl-CoA via the acetyl-CoA carboxylase activity [47]. As in the previous strain, the naringenin BGC was integrated into the chromosomal φC31 attB site, giving rise the *S. albidoflavus* UO-FLAV-004-NAR strain.

**Fig. 2:**
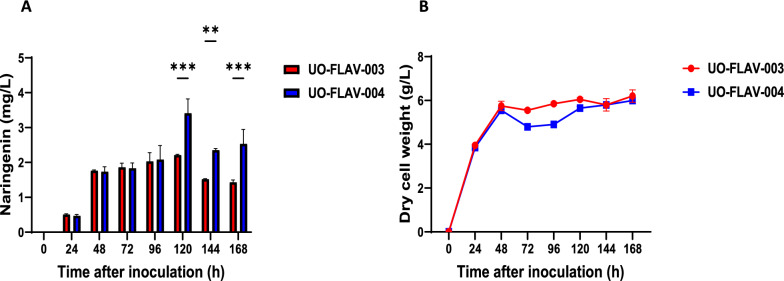
Comparison of naringenin titers (**A**) and biomass (**B**) between the strains *S. albidoflavus* UO-FLAV-003-NAR and *S. albidoflavus* UO-FLAV-004-NAR at different time points after inoculation

Both strains, *S. albidoflavus* UO-FLAV-003-NAR and *S. albidoflavus* UO-FLAV-004-NAR were cultivated in triplicate in NL333 medium. The biosynthesis of naringenin was measured at 24, 48, 72, 96, 120, 144 and 168 h after inoculation (Fig. 2A). The naringenin production titers in the *S. albidoflavus* UO-FLAV-003-NAR strain reached a maximum of 2.2 mg/L at 120 h (Fig. 2). The best production titers in the parental strain *S. albidoflavus* UO-FLAV-002-NAR were 1.63 mg/L at 48 h [45], which is a similar level of production. In the case of the *S. albidoflavus* UO-FLAV-004-NAR strain, it produced a maximum of 3.4 mg/L at 120 h, which supposes a significant 1.6-fold increase with respect to the strain *S. albidoflavus* UO-FLAV-003-NAR at the same time point, while no significant differences were observed with *S. albidoflavus* UO-FLAV-003-NAR at 48 h. The biomass was also monitored at the same time points to discard a possible misbalance in the growth rate between both strains that could lead a production improvement (Fig. 2B). No significant differences were observed in the biomass, supporting that the deletion of the paulomycins BGC was mainly responsible for the observed naringenin production increase.

### Heterologous biosynthesis of sakuranetin

The methylated flavanone sakuranetin is generated due to the action of a flavonoid 7-*O*-methyltransferase (7OMT), using as substrate the flavonoid precursor naringenin and SAM as the methyl group donor. In this work, the gene selected for this purpose was *OsNOMT* from *Oryza sativa* [48]*.*This plant gene was optimized at the codon usage level for its expression in *S. albidoflavus* and it was assembled under the control of the SF14 promoter [49] (see “Materials and Methods” section). The plasmid pSEVAUO-M21104-OsNOMT was integrated into the chromosomal φBT1 attB site of the *S. albidoflavus* UO-FLAV-004-NAR strain, generating the *S. albidoflavus* UO-FLAV-004-SAK strain.

**Fig. 3:**
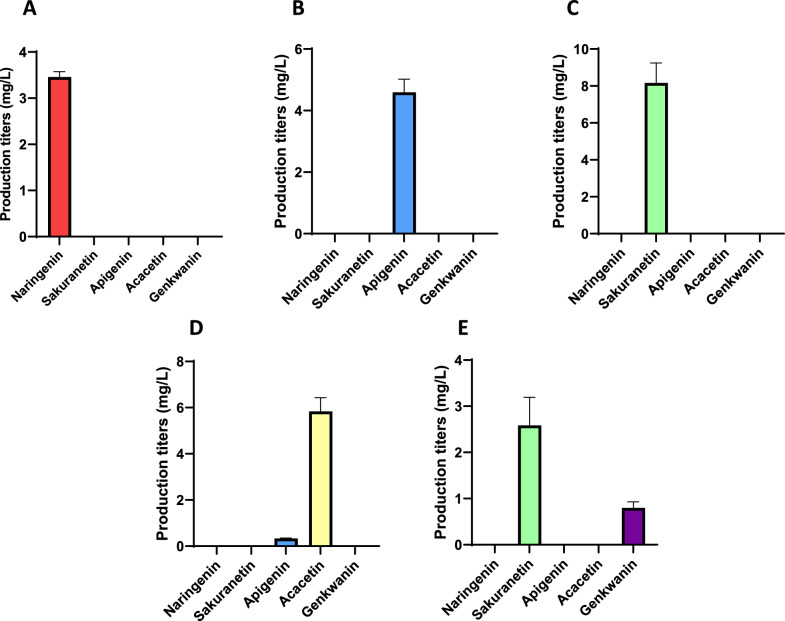
Production titers of different flavonoids produced in the different flavonoid producer strains of *S. albidoflavus* UO-FLAV-004. **A** *S. albidoflavus* UO-FLAV-004-NAR; **B** *S. albidoflavus* UO-FLAV-004-API; **C** *S. albidoflavus* UO-FLAV-004-SAK; **D** *S. albidoflavus* UO-FLAV-004-ACA; **E** *S. albidoflavus* UO-FLAV-004-GNK

Cultures of the strain *S. albidoflavus* UO-FLAV-004-SAK, and the control strain *S. albidoflavus* UO-FLAV-004-NAR, were carried out in NL333 medium and analyzed by HPLC-DAD chromatography to identify and quantify the final production of naringenin and sakuranetin. Both naringenin and sakuranetin were quantified using commercial pure standards. The naringenin production titers in the control strain *S. albidoflavus* UO-FLAV-004-NAR reached 3.5 mg/L (Fig. 3A and Additional file 1: Fig. S1), an amount that agrees with the production experiment discussed in the previous section. The sakuranetin titers in the *S. albidoflavus* UO-FLAV-004-SAK strain were 8.2 mg/L (Fig. 3C and Additional file 1: Fig. S1), while the precursor naringenin was not detected, indicating a total conversion (Additional file 2: Fig. S2).

### Heterologous biosynthesis of acacetin

Acacetin is a methylated form of the flavone apigenin. For its biosynthesis, two extra enzymatic activities are needed, acting on the naringenin intermediate. First, the flavone synthase (FNS) carries out a dehydration in the ring C, generating a double bond and giving rise to apigenin from naringenin (Fig. 1) [38]. Then, a 4′-O-methyltransferase (4′OMT) catalyzes the addition of a methyl group in the 4’ position of the ring B, giving rise to acacetin. The selected 4’OMT in this work was *Pa4*′*OMT* from *Plagiochasma appendiculatum* [50]. Both, FNS and 4’OMT coding genes were assembled separately under the control of the SF14 promoter and then assembled to generate the plasmid pSEVAUO-M21503-ACA, which was integrated into the chromosomal φBT1 attB site of the *S. albidoflavus* UO-FLAV-004-NAR strain, giving rise the *S. albidoflavus* UO-FLAV-004-ACA strain. We also generated an apigenin producing strain by the integration of the plasmid pSEVAUO-M21202-FNS1 into the chromosomal φBT1 integration site of the *S. albidoflavus* UO-FLAV-004-NAR strain, yielding the strain *S. albidoflavus* UO-FLAV-004-API, which produces 4.6 mg/L of apigenin (Fig. 3B and Additional file 1: Fig. S1). The retention time of apigenin in the chromatography is practically the same as that of naringenin (Additional file 3: Fig. S3A), but both UV absorption spectra are different and allows them to be differentiated (Additional file 3: Fig. S3B and S3C). To ensure that the detected production is only apigenin, further analyses with HPLC-HRESIMS were carried out, and an intense signal of *m/z* 269.0455 [M-H]^−^ (calculated for C_15_H_10_O_5_, corresponding to the M signal from the isotopic cluster of apigenin) was detected, and also a small signal of *m/z* 271.0612 [M-H]^−^ (calculated for C_15_H_12_O_5_, corresponding to the M + 2 signal from the isotopic cluster of apigenin) was also detected (Additional file 3: Fig. S3D). The signals M + 1 and M + 2 from the isotopic cluster of naringenin are not detected, indicating that naringenin is not accumulated in this strain.

The apigenin producer strain was made as a control for the biosynthesis of the apigenin methyl derivatives described in this work. These two strains were cultivated in NL333 medium, and the acacetin production titer reached 5.8 mg/L in the *S. albidoflavus* UO-FLAV-004-ACA extract (Fig. 3D and Additional file 1: S1). No remaining naringenin and a small peak of apigenin were detected in the *S. albidoflavus* UO-FLAV-004-ACA extract (Additional file 4: Fig. S4). The accumulated amount of apigenin in the acacetin producer strain reached 0.3 mg/L (Fig. 3D and Additional file 1: Fig. S1). Only apigenin and not acacetin were detected in the control strain (Additional file 2: Fig. S2).

### Heterologous biosynthesis of genkwanin

The biosynthesis of genkwanin is produced by a methylation in the position 7 of the ring A of apigenin, carried out by a 7-O-methyltransferase. The pSEVAUO-M21104-OsNOMT and pSEVAUO-M21202-FNS1 recombinant plasmids were used to generate the pSEVAUO-M21503-GNK final vector (see “Materials and Methods” section), and in the same manner as in the previous strains, this final recombinant vector was transformed in *S. albidoflavus* UO-FLAV-004-NAR and integrated into the chromosomal φBT1 attB site, giving rise to the *S. albidoflavus* UO-FLAV-004-GNK strain. This strain was able to produce genkwanin with a production titer of 0.8 mg/L (Fig. 3E and Additional file 1: Fig. S1), which was a lower amount than those observed in the case of acacetin or sakuranetin producing strains. The reason for such a low production titer of genkwanin is that this strain is also producing sakuranetin in higher amount (2.6 mg/L), which represents a bottleneck in the genkwanin biosynthesis due to the higher affinity of the 7OMT enzyme for naringenin than for apigenin. No remaining naringenin or apigenin were detected in these extracts (Additional file 5: Fig. S5).

### Feeding experiments and enhancement of the biosynthesis of genkwanin using co-cultures

To check the substrate flexibility of the involved enzymes, with the aim of setting up a strategy to increase the genkwanin titers, the strains *S. albidoflavus* UO-FLAV-004-FNS1, harboring only the gene that encodes FNS1*,* and *S. albidoflavus* UO-FLAV-004-OsNOMT, harboring only the gene that encodes OsNOMT, were fed with 0.1 mM sakuranetin and 0.1 mM apigenin, respectively. No genkwanin was detected in the sakuranetin feeding experiment (Additional file 6: Fig. S6), indicating that FNS1 enzyme does not accept this flavonoid as substrate. However, a peak of genkwanin was detected after feeding with apigenin (Additional file 7: Fig. S7), indicating that the OsNOMT enzyme can almost totally convert apigenin in vivo into genkwanin (more than 95%), while in vitro the conversion rate is 61% [48].

**Fig. 4:**
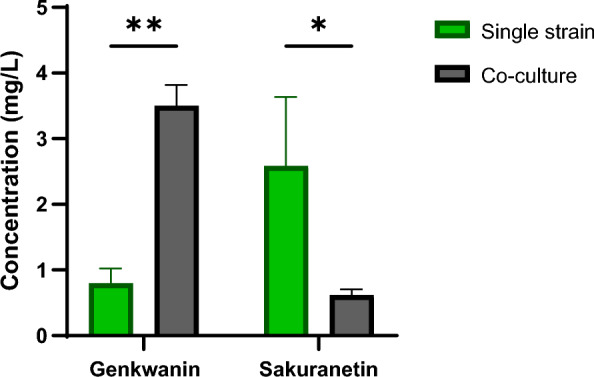
Production titers of sakuranetin and genkwanin when produced in a single strain (*S. albidoflavus* UO-FLAV-004-GNK, green color) or co-cultures (*S. albidoflavus* UO-FLAV-004-API plus *S. albidoflavus* UO-FLAV-004-OsNOMT strains, gray color)

These results suggest that a co-culture between *S. albidoflavus* UO-FLAV-004-API and *S. albidoflavus* UO-FLAV-004-OsNOMT could be a good alternative to alleviate the direct naringenin deviation towards sakuranetin in the biosynthesis of genkwanin, since the strain *S. albidoflavus* UO-FLAV-004-API produces apigenin with high efficiency (4.6 mg/L), which implies a good availability of this flavonoid for its final conversion to genkwanin. The co-cultures were performed as described in “Materials and Methods” section, using as control the *S. albidoflavus* UO-FLAV-004-API strain in co-culture with the strain *S. albidoflavus* UO-FLAV-004, resulting in the production of apigenin. The co-culture between *S. albidoflavus* UO-FLAV-004-API and *S. albidoflavus* UO-FLAV-004-OsNOMT strains resulted in the biosynthesis of genkwanin and a small amount of sakuranetin as a shunt product (Additional file 8: Fig. S8). The titers of genkwanin in these experiments were 3.5 mg/L, which represents a 4.4-fold increase in comparison with the single strain initial option. On the other hand, the biosynthesis of sakuranetin dropped to 0.6 mg/L (Fig. 4), reverting the scenario observed in the strain *S. albidoflavus* UO-FLAV-004-GNK.

## Discussion

In this work, two BGCs (coding for the biosynthesis of PTMs and paulomycins) were removed from the chromosome of *S. albidoflavus* to try to increase cytosolic pools of malonyl-CoA and L-Tyr, the two building blocks for flavonoid heterologous biosynthesis in this bacterium*.* Several other BGCs in this species have been previously removed from the chromosome of this bacterium by other authors, to facilitate the biosynthesis of other heterologous compounds [51].

To remove the PTM BGC from the genome of the strain *S. albidoflavus* UO-FLAV-002, the plasmid pSEVAUO-C41012-BGC2 was used (Additional file 9: Fig. S9). This BGC consumes malonyl-CoA, and therefore the mutant strain *S. albidoflavus* UO-FLAV-003 should be a better flavonoid producer due to higher bioavailability of this building block. However, the naringenin titers in the *S. albidoflavus* UO-FLAV-003-NAR strain were similar to those observed in its parental *S. albidoflavus* UO-FLAV-002-NAR strain [45]. The absence of a clear effect over the biosynthesis of flavonoids could be due to the non-constitutive expression of the PTM BGC in *S. albidoflavus* under laboratory cultivation conditions*,* as happens in other hosts [52].

The pSEVAUO-C41012-BGC5 plasmid was used for removing the paulomycin BCG from the *S. albidoflavus* UO-FLAV-003 chromosome (Additional file 10: Fig. S10). The biosynthesis of these glycosylated antibiotics consumes chorismate and acetyl-CoA [46]. Chorismate is a precursor of L-tyrosine, the first building block involved in flavonoid biosynthesis in our heterologous system, while acetyl-CoA is necessary for malonyl-CoA biosynthesis through the acetyl-CoA carboxylase complex. The deletion of this BGC in the *S. albidoflavus* UO-FLAV-004-NAR strain led to a 55% increase in naringenin titers in comparison with the strain *S. albidoflavus* UO-FLAV-003-NAR, and this positive effect is not due to a biomass misbalance (Fig. 2B), but most probably to the fact that the paulomycin BGC is normally active in *S. albidoflavus* under these cultivation conditions. The final products of this metabolic pathway, paulomenol A and B [46], can be detected in cellular extracts of the *S. albidoflavus* J1074 wild type strain but not in the *S. albidoflavus* UO-FLAV-004 strain using HPLC-HRESIMS (Additional file 11: Fig. S11). In this manner, after the paulomycin BGC deletion, the flavonoid precursors cytosolic pool was increased. These results highlight the importance of tailor-made metabolic engineering for enhancing the biosynthesis of bioactive compounds.

In previous works, our research group has achieved the production of several flavonoids derived from naringenin chalcone in *S. albidoflavus* [38, 53–55]. To our knowledge, this study describes for first time the biosynthesis of O’-methylated flavonoids in actinomycetes, and in general in Gram-positive bacteria, enhancing their final production titers by metabolic engineering. Regarding this, the biosynthesis of sakuranetin was previously achieved in *E. coli* [36] and using *E. coli* co-cultures [56]. Other authors also achieved its production in *E. coli* under feeding with precursors [57]. In this study, sakuranetin was produced by integrating in the chromosomal φBT1 attb site of the strain *S. albidoflavus* UO-FLAV-004-NAR the *OsNOMT* gene, coding for an O-methyltransferase necessary to add a methyl group in the position 7 at ring A of naringenin [58]. When comparing the naringenin levels in the *S. albidoflavus* UO-FLAV-004-NAR strain with sakuranetin in the *S. albidoflavus* UO-FLAV-004-SAK strain, the titers of the 7-O-methylated derivative were higher than those of naringenin (Fig. 3). This striking result can be explained since it has been reported that different shunt products are generated from naringenin, such as bisnoryangonin (generated as a derailment shunt product by the CHS enzyme after just two malonyl-CoA condensations) and p-coumaroyltriacetic acid lactone (generated as a derailment shunt product by the CHS enzyme after three malonyl-CoA condensations) [59, 60].

In the case of acacetin, its biosynthesis has been achieved from simple carbon sources using the Gram-negative *E. coli* in co-cultures (two or three different strains), each of them with a part of the biosynthetic pathway, reaching titers of 20.3 mg/L [61]. In this study, we report the first biosynthesis of acacetin culturing a single strain, with final production titers of 5.8 mg/L [61]. In this *S. albidoflavus* UO-FLAV-004-ACA strain, the genes *PcFNS1* and *Pa4*′*OMT,* coding for FNS1 and 4′-O-methyltransferase respectively, were integrated into the chromosomal φBT1 attb site of the *S. albidoflavus* UO-FLAV-004-NAR strain. *Pa4*′*OMT* has been used before as an apigenin 4′-O-methyltransferase in *E. coli,* where this enzyme yields 88.8 µM acacetin after feeding with 100 µM apigenin precursor [50]. In *S. albidoflavus* UO-FLAV-004-ACA, only 0.3 mg/L of apigenin were accumulated after cultivation, and no naringenin derivatives were detected, since the Pa4’OMT enzyme does not recognize this flavanone as a substrate [50].

Finally, the heterologous biosynthesis of genkwanin has also been reported in *E. coli* [62] using a 7-O-methyltransferase from *Populus deltoides* [63]. To achieve in *S. albidoflavus* the biosynthesis of genkwanin, we have combined the *OsNOMT* gene used for the biosynthesis of sakuranetin with the *PcFNS1* gene used for the biosynthesis of apigenin. Both enzymes were cloned together in the plasmid pSEVAUO-M21503-GNK and integrated in the chromosomal φBT1 attb site of the *S. albidoflavus* UO-FLAV-004-NAR strain. The OsNOMT enzyme can use apigenin as substrate, although it prefers naringenin [48]. The success in this experiment depends on the affinity of the PcFNS1 for naringenin, because the OsNOMT methyltransferase could use naringenin as a substrate and then give rise to sakuranetin. On the other hand, PcFNS1 could act on sakuranetin to generate genkwanin, but this activity was not observed. After the cultivation of the strain *S. albidoflavus* UO-FLAV-004-GNK, two peaks were detected in the HPLC chromatograms of culture extracts (Additional file 5: Fig. S5), corresponding to sakuranetin and genkwanin. However, the sakuranetin amount (2.6 mg/L) was significantly higher than that of genkwanin (0.8 mg/L).

To determine if genkwanin comes from the methylation of apigenin by OsNOMT, or from a double bond formation in the ring C of sakuranetin by PcFNS1*,* we decided to try the *OsNOMT* and *PcFNS1* genes separately. Both genes were integrated in the chromosomal φBT1 attb site of *S. albidoflavus* UO-FLAV-004. The strain *S. albidoflavus* UO-FLAV-004-OsNOMT was fed with apigenin, and this substrate was converted totally into genkwanin after five days of cultivation. The strain *S. albidoflavus* UO-FLAV-004-FNS1 was fed with sakuranetin, but in these cultures no genkwanin was detected, only an accumulation of sakuranetin. These results indicate that OsNOMT is using naringenin as a substrate in a faster way than PcFNS1 in the *S. albidoflavus* UO-FLAV-004-GNK strain, giving rise to sakuranetin and generating a bottleneck in the biosynthesis of genkwanin.

To increase the genkwanin titers, a co-culture strategy was developed, performing two cultivations separately. On one side, *S. albidoflavus* UO-FLAV-004-API was cultivated during four days for the accumulation of apigenin, and on the other hand, *S. albidoflavus* UO-FLAV-004-OsNOMT was cultivated at the same time. At the end of day four, half of each culture was brought together in a new flask and incubated two more days. In this new scenario, the genkwanin titers raised to 3.5 mg/L, while sakuranetin titers dropped to 0.6 mg/L. These results indicate that co-culture experiments can be useful not only to split long pathways and facilitate the flavonoid production in *S. albidoflavus*, but also in cases where the enzymes involved in the biosynthesis have substrate flexibility and different affinities over distinct precursors.

## Conclusions

Metabolic engineering is a key strategy to increase the biosynthesis of flavonoids in heterologous hosts, such as actinomycetes. Deletion of BGCs encoding compounds that are produced under laboratory cultivation conditions and consuming common precursors shared with the flavonoid biosynthetic pathway, such as chorismate or malonyl-CoA, is a good strategy to boost the final flavonoid titers in these bacterial factories. In this work, *S. albidoflavus* has been proven as a good platform for the biosynthesis of the methylated flavonoids sakuranetin, acacetin, and genkwanin, which are bioactives with high interest at the pharmaceutical and nutraceutical levels. Finally, the strategy of establishing co-cultures has been able to avoid by-products derived from the substrate flexibility of enzymes involved in the biosynthetic pathway of interest. However, it could be economically limiting at industrial level due to the high cost of building two biomass pools to produce one compound.

## Materials and methods

### Reagents and biochemicals

All solvents used for solid phase extraction and HPLC-DAD analysis were LC-MS grade from either Sigma-Aldrich (Madrid, Spain) or VWR Chemicals (Barcelona, Spain). Apigenin and acacetin were purchased from Sigma-Aldrich, whilst naringenin, sakuranetin and genkwanin were provided by Extrasynthese (Genay, France).

### Genes and enzymes

Restriction enzymes and T4 DNA ligase were purchased from Thermo Fisher Scientific (Madrid, Spain). Herculase II Fusion DNA polymerase was purchased from Agilent Technologies (Madrid, Spain), Terra PCR Direct polymerase from Takara (Saint-Germain-en-Laye, France), and NEBuilder® HiFi DNA Assembly Master Mix from New England BioLabs (MA, USA). Synthetic genes for the following ORFs were synthesized by Integrated DNA Technologies (IDT, NJ, USA after codon optimization: *PcFNS1* from *Petroselinum crispum* (Genbank accession no. OR327443), *OsNOMT* from *Oryza sativa* (Genbank accession no. OR327442), and *Pa4’OMT* from *Plagiochasma appendiculatum* (Genbank accession no. OR327441). The primers used for the generation and checking of the *S. albidoflavus* UO-FLAV-003 and *S. albidoflavus* UO-FLAV-004 strains are listed in Additional file 12: Table S1.

### Construction of pSEVAUO-C41012-BGC2 and pSEVAUO-C41012-BGC5 plasmids for genome editing

All the plasmids in this study are listed in Table 1. To generate CRISPR-Cas9 based plasmids for the deletion of endogenous BGCs, a protospacer of 20 bp for each one of these native BGCs was designed and cloned into the pSEVAUO-C41012 vector [45] using a Golden Gate reaction, generating pSEVAUO-C41012-Spacer-BGC2 (chromosomal position 244,648–244,667) and pSEVAUO-C41012-Spacer-BGC5 (chromosomal position 690,078–690,098). Two homologous arms flanking each of the two biosynthetic gene clusters of interest (PTMs and paulomycins) were amplified from the *S. albidoflavus* genome using HerculaseII Fusion DNA polymerase and cloned into the pSEVA88c1 vector [45] by Gibson assembly, giving rise to pSEVA88c1-BGC2 (flanking homologous arms include chromosomal regions 254,894–257,085 and 237,272–239,274) and pSEVA88c1-BGC5 (flanking homologous arms include chromosomal regions 720,066–720,095and 674,513–674,543), respectively. The corresponding homologous arms were then cloned into pSEVAUO-C41012-Spacer-BGC2 and pSEVAUO-C41012-Spacer-BGC5 plasmids, using the restriction enzymes *Pac*I and *Spe*I and the T4 DNA ligase, leading to the generation of pSEVAUO-C41012-BGC2 and pSEVAUO-C41012-BGC5 final plasmids for these genome editions.

**Table 1:** Plasmids and strains used in this study

	Description	Source
*Plasmids*		
pSEVA88c1	Replicative shuttle vector	[45]
pSEVA88c1-BGC2	pSEVA88c1 harboring homologous arms for BGC2 deletion	This study
pSEVA88c1-BGC5	pSEVA88c1 harboring homologous arms for BGC5 deletion	This study
pSEVAUO-C42012	Replicative shuttle vector harboring the nuclease cas9	[42]
pSEVAUO-C42012-Spacer-BGC2	pSEVAUO-C42012 harboring the protospacer for BGC2 deletion	This study
pSEVAUO-C42012-Spacer-BGC5	pSEVAUO-C42012 harboring the protospacer for BGC2 deletion	This study
pSEVAUO-C42012-BGC2	pSEVAUO-C42012-Spacer-BGC2 harboring homologous arms for BGC2 deletion	This study
pSEVAUO-C42012-BGC5	pSEVAUO-C42012-Spacer-BGC5 harboring homologous arms for BGC5 deletion	This study
pSEVAUO-M11701-NarBGC	Level 2 MoClo plasmid harboring *TAL, 4CL, CHS* and *CHI*	[42]
PCR-Blunt II-TOPO	Replicative blunt DNA cloning vector	Invitrogen
PCR-Blunt II-TOPO-OsNOMT	PCR-Blunt II-TOPO harboring *OsNOMT*	This study
pSEVA181SF14	Source of *SF14* (Level 0 MoClo)	EXPLORA
pSEVA181RiboJ-RBS	Source of *RiboJ-RBS* (Level 0 MoClo)	EXPLORA
pIDTSMARTttsbib	Source of *ttsbib* (Level 0 MoClo)	IDT
pSEVAUO-M21104	Level 1 MoClo receptor	[42]
pSEVAUO-M21104-OsNOMT	Level 1 MoClo harboring *OsNOMT*	This study
PCR-Blunt II-TOPO-Pa4’OMT	PCR-Blunt II-TOPO harboring *Pa4’OMT*	This study
PCR-Blunt II-TOPO-FNS1	PCR-Blunt II-TOPO harboring *FNS1*	This study
pSEVAUO-M21102	Level 1 MoClo receptor	[42]
pSEVAUO-M21202-FNS1	Level 1 MoClo harboring *FNS1*	This study
pSEVAUO-M21202	Level 1 MoClo receptor	[42]
pSEVAUO-M21102-Pa’4OMT	Level 1 MoClo harboring *Pa4’OMT*	This study
pSEVAUO-M21503	Level 2 MoClo receptor	[42]
pSEVAUO-M21503-ACA	Level 2 MoClo plasmid harboring *FNS1* and *Pa4’OMT*	This study
pSEVAUO-M21503-GNK	Level 2 MoClo plasmid harboring *FNS1* and *OsNOMT*	This study
*Strains*		
*E. coli* TOP10	Strain used for routine subcloning	Invitrogen
*E. coli* ET12567/pUZ8002	Strain used for conjugation	Life Science
UO-FLAV-002	*S. albidoflavus* strain used for BGC2 deletion	[42]
UO-FLAV-003	UO-FLAV-002 lacking the BGC2	This study
UO-FLAV-004	UO-FLAV-003 lacking the BGC5	This study
UO-FLAV-003-NAR	UO-FLAV-003 harboring *TAL, 4CL, CHS* and *CHI*	This study
UO-FLAV-004-NAR	UO-FLAV-004 harboring *TAL, 4CL, CHS* and *CHI*	This study
UO-FLAV-004-API	UO-FLAV-004 harboring *TAL, 4CL, CHS*, *CHI* and *FNS1*	This study
UO-FLAV-004-SAK	UO-FLAV-004 harboring *TAL, 4CL, CHS*, *CHI* and *OsNOMT*	This study
UO-FLAV-004-ACA	UO-FLAV-004 harboring *TAL, 4CL, CHS*, *CHI, FNS1* and *Pa4’OMT*	This study
UO-FLAV-004-GNK	UO-FLAV-004 harboring *TAL, 4CL, CHS*, *CHI, FNS1* and *OsNOMT*	This study
UO-FLAV-004-OsNOMT	UO-FLAV-004 harboring *OsNOMT*	This study
UO-FLAV-004-FNS1	UO-FLAV-004 harboring *FNS1*	This study

### Construction of pSEVAUO-M21104-OsNOMT plasmid

The *OsNOMT* gene, designed for MoClo assembly [64], was cloned into the PCR-Blunt II-TOPO vector, giving rise the PCR-Blunt II-TOPO-OsNOMT. The plasmid pSEVAUO-M21104-OsNOMT was then assembled in a level 1 MoClo reaction from the level 0 plasmids pSEVA181SF14, pSEVA181RiboJ-RBS, pIDTSMARTttsbib [45], PCR-Blunt II-TOPO-OsNOMT (this study) and the level 1 receptor pSEVAUO-M21104 [45].

### Construction of pSEVAUO-M21503-ACA plasmid

The *Pa4’OMT* gene, designed for MoClo assembly, was cloned into the PCR-Blunt II-TOPO vector giving rise the PCR-Blunt II-TOPO-FNS1. The level 1 plasmid pSEVAUO-M21202-FNS1 was generated from the level 0 plasmids pSEVA181SF14, pSEVA181RiboJ-RBS, pIDTSMARTttsbib, PCR-Blunt II-TOPO-Pa4’OMT (this work) and the level 1 receptor pSEVAUO-M21202.

The *PcFNS1* gene was cloned in the same way than the previous ORFs to generate the PCR-Blunt II-TOPO-FNS1 recombinant plasmid. The level 1 plasmid pSEVAUO-M21102-Pa’4OMT was assembled from the level 0 plasmids pSEVA181SF14, pSEVA181RiboJ-RBS, pIDTSMARTttsbib, PCR-Blunt II-TOPO-FNS1 (this work) and the level 1 receptor pSEVAUO-M21102 [45]. Finally, the pSEVAUO-M21503-ACA was assembled in a level 2 MoClo reaction using the level 1 plasmids pSEVAUO-M21202-FNS1 and pSEVAUO-M21102-Pa’4OMT, and the level 2 receptor pSEVAUO-M21503 [45].

### Construction of pSEVAUO-M21503-GNK plasmid

The plasmid pSEVAUO-M21503-GNK was assembled in a level 2 MoClo reaction using the level 1 plasmids pSEVAUO-M21104-OsNOMT, pSEVAUO-M21202-FNS1, and the level 2 receptor pSEVAUO-M21503.

### Bacterial strains and culture conditions

All strains in this study are listed in Table 1. *Escherichia coli* TOP10 (Invitrogen, Waltham, MA, USA) was used for routine subcloning. *E. coli* ET12567/pUZ8002 (Thermo Fisher Scientific, Madrid, Spain) was used for conjugation. All the *S. albidoflavus* strains presented in this work have been generated by bacterial conjugation using the previously mentioned *E. coli* ET12567/pUZ8002 strain. The new strains were confirmed by antibiotic resistance and further corroborated with the biosynthesis of the desired compounds. The strain *S. albidoflavus* UO-FLAV-002 [45], a mutant of the *S. albidoflavus* J1074 that lacks the chromosomal pseudo-attB site for the ΦC31 recombination system [65] and the native biosynthetic gene clusters of candicidins and antimycins, was used for further metabolic engineering. The strain *S. albidoflavus* UO-FLAV-003 (this study), a mutant of *S. albidoflavus* UO-FLAV-002 that lacks the native biosynthetic gene cluster of PTMs, was generated using the CRISPR based plasmid pSEVAUO-C41012-BGC2 and transformed with pSEVAUO-M11701-Nar [45] that directs the biosynthesis of naringenin, giving rise *S. albidoflavus* UO-FLAV-003-NAR. The strain *S. albidoflavus* UO-FLAV-004 (this study)*,* a mutant of *S. albidoflavus* UO-FLAV-003 that lacks the biosynthetic gene cluster of paulomycins, was generated using the CRISPR based plasmid pSEVAUO-C41012-BGC2 and transformed with the plasmid pSEVAUO-M11701-Nar, giving rise to the strain *S. albidoflavus* UO-FLAV-004-NAR. The *S. albidoflavus* UO-FLAV-004-NAR strain was transformed with several plasmids directing the biosynthesis of different flavonoids, generating the strains *S. albidoflavus* UO-FLAV-004-API (apigenin producer), *S. albidoflavus* UO-FLAV-004-SAK (sakuranetin producer), *S. albidoflavus* UO-FLAV-004-ACA (acacetin producer), and *S. albidoflavus* UO-FLAV-004-GNK (genkwanin producer). The plasmids pSEVAUO-M21202-FNS1 and pSEVAUO-M21104-OsNOMT were also transformed in *S. albidoflavus* UO-FLAV-004 for substrate specificity assays, generating *S. albidoflavus* UO-FLAV-004-FNS1, and *S. albidoflavus* UO-FLAV-004-OsNOMT, respectively.

*E. coli* strains were grown in tryptic soy broth (TSB, VWR, Barcelona, Spain) or on TSB agar plates, supplemented with the corresponding antibiotic (ampicillin 100 µg/mL, Sigma Aldrich (Madrid, Spain); apramycin 100 µg/mL, Thermo Fisher Scientific (MA, USA); gentamycin 50 µg/mL, Thermo Fisher Scientific (MA, USA) and X-gal (AppliChem, Darmstadt, Germany) when blue-white selection was needed. *S. albidoflavus* was grown at 30 ºC in yeast extract-malt extract (YEME) 17% (w/v) sucrose for the preparation of protoplasts, and MA medium was used for conjugation experiments [66]. This species was grown to obtain spores on Bennet medium [67] and supplemented with the corresponding antibiotics, when necessary (thiostrepton 50 µg/mL, Cayman Chemical, MI, USA, or apramycin 50 µg/mL). For flavonoid production, *S. albidoflavus* spores were quantified, and an inoculum of 10^6^ spores/mL was performed in triplicate in shake flasks with 25 mL of NL333 medium [68] and incubated during 120 h at 30 ºC and 250 rpm.

The *S. albidoflavus* co-cultures were performed for 6 days. During the 4 first days the strains were grown separately as described before. At the end of day 4, 12.5 mL of each culture were brought together in a new flask and incubated 2 more days under the same conditions.

### Flavonoid extraction, HPLC-DAD and HPLC-HRESIMS analysis

Spores from the different *S. albidoflavus* strains were incubated as described before in NL333 culture medium (10^6^ spores/mL). Flavonoids were obtained by an organic extraction with acetone (cellular pellet) and ethyl acetate (culture supernatant). A sample of 1 mL was taken from the flasks and centrifuged at 12,000 rpm for 1 min to separate the culture supernatant from the pellet. The pellet was extracted with 1 mL of acetone using vortex for 1 h. The supernatant was extracted with 800 µL of ethyl acetate under vortexing for 10 min. Both pellet and supernatant extractions were centrifuged for 1 min at 12,000 rpm and the organic fractions were mixed and dried in a speed-vac. A second extraction was performed using 800 µL ethyl acetate on both the cellular pellet and the supernatant, using vortex, as described before. Finally, both extractions were mixed over the dry extract obtained in the first extraction and dried in a speed-vac.

For the identification of flavonoids using HPLC-DAD, the final dry extract obtained from each cultivation condition was dissolved in 100 µL DMSO/MeOH 1:1 (v/v), and the samples were centrifuged prior to the injection in the equipment. The HPLC separation was performed in a HPLC (1260 Infinity, Agilent Technologies, Madrid, Spain) equipped with an analytical column Pursuit XRs C18 (50 × 4.0 mm, 5 μm, Agilent Technologies, Madrid, Spain). HPLC gradient was made with analytical grade solvent B (acetonitrile 100% (VWR, Spain)), and water as solvents (1 mL/min flow rate). All solvents contained 0.1% formic acid. Samples were eluted using this HPLC program: 10% to 40% acetonitrile at 0–10 min, 40%-50% acetonitrile at 10–30 min, 50%-100% acetonitrile at 30–40 min, and 100%-10% acetonitrile at 40–50 min. Detection and spectral characterization of peaks were carried out with a photodiode array detector and the analysis was performed with the Data Analysis 4.3 software (Bruker). All chromatograms were extracted at 280 nm. The column temperature was set to 30 ºC. The flavonoids were identified using authentic commercial standards and quantified by comparing the peak area with that of a known amount of an authentic compound through a calibration curve. The production titers are given in mg/L, and the mean value was calculated from three biological replicates.

For the identification of paulomenols using HPLC-HRESIMS, the samples were extracted as described above. Separation was performed in a UPLC system (Dionex Ultimate 3000, Thermo Scientific, Madrid, Spain) equipped with an analytical RP-18 HPLC column (50 9 2.1 mm, Zorbax® Eclipse Plus, 1.8 µm, Agilent Technologies, Madrid, Spain) heated to 30 °C, and a combination of distilled water (mobile phase A) and acetonitrile (mobile phase B), both acidified with 0.1% (v/v) of formic acid, was used. The analytes were eluted at a flow rate of 0.25 ml min_1 in a 10–100% (v/v) gradient of acetonitrile under the following conditions: 0–1 min (10% B), 1–4 min (10–35% B), 4–5 min (35% B), 5–8 min (35–100% B), 8–10 min (100% B), 10–11 min (100–10% B) and 11–15 min (10% B). The column effluent was directed to electrospray ionization mass spectrometry analysis (HPLC-ESI-MS) using an ESI-UHR-Qq-TOF Impact II spectrometer (Bruker Española SA, Madrid, Spain) which acquired data in the negative ion mode, with a *m/z* range from 40 to 2000 Da. Data were analyzed using Compass Data Analysis 4.3 (Bruker). The obtained base peaks chromatograms (BPCs) were extracted for the deprotonated ions of a set of flavonoids with a mass error range of 0.005 mmu (milli mass units), and the obtained EICs (extracted ion chromatograms) were compared with authentic commercial standards.

### Statistical analysis

Two-way ANOVA (analysis of variance Sidak's multiple comparisons test) was used for testing the differences in the biosynthesis of naringenin among the strains *S. albidoflavus* UO-FLAV-003-NAR and *S. albidoflavus* UO-FLAV-004-NAR, and the biosynthesis of genkwanin among the strain *S. albidoflavus* UO-FLAV-004-GNK and the co-culture established with the strains *S. albidoflavus* UO-FLAV-004-API and *S. albidoflavus* UO-FLAV-004-OsNOMT. Graphic representation of the different data generated was carried out using GraphPad Prism software (version 9.0.2, GraphPad Software, San Diego, CA, USA), and considering a *p* value < 0.05 as statistically significant (*p < 0.05; **p < 0.005; ***p < 0.0005; ****p < 0.0001).

### Supplementary Information

**Additional file 1: Figure S1.** HPLC-DAD chromatograms of the strains *S. albidoflavus* UO-FLAV-004-NAR (red), *S. albidoflavus* UO-FLAV-004-SAK (green), *S. albidoflavus* UO-FLAV-004-API (blue), *S. albidoflavus* UO-FLAV-004-ACA (black), *S. albidoflavus* UO-FLAV-004-GNK (orange). Naringenin (NAR); Sakuranetin (SAK), Apigenin (API); Acacetin (ACA); Genkwanin (GNK).

**Additional file 2: Figure S2.** HPLC-DAD chromatograms of *S. albidoflavus* UO-FLAV-004-NAR (red) and *S. albidoflavus* UO-FLAV-004-SAK (green). Naringenin (NAR); Sakuranetin (SAK).

**Additional file 3: Figure S3.** A) HPLC-DAD chromatograms of *S. albidoflavus* UO-FLAV-004-NAR (red) and *S. albidoflavus* UO-FLAV-004-API (blue) showing naringenin and apigenin production, respectively. B) Absorption spectrum of naringenin pure standard in a concentration of 500 µM. C) Absorption spectrum of apigenin pure standard in a concentration of 500 µM. D) Extracted BPCs of apigenin (*m/z* 269.0455 [M-H]^−^, blue color) and naringenin (*m/z* 271.0612 ± [M-H]^−^, black color) from a sample of the strain *S. albidoflavus* UO-FLAV-004-API, where only signals from the apigenin isotopic cluster are detected.

**Additional file 4: Figure S4.** HPLC-DAD chromatograms of *S. albidoflavus* UO-FLAV-004-API (red) and *S. albidoflavus* UO-FLAV-004-ACA (black). Apigenin (API); Acacetin (ACA).

**Additional file 5: Figure S5.** HPLC-DAD chromatograms of *S. albidoflavus* UO-FLAV-004-API (red) and *S. albidoflavus* UO-FLAV-004 GENK (blue). Apigenin (API); Sakuranetin (SAK); Genkwanin (GNK).

**Additional file 6: Figure S6.** HPLC-DAD chromatograms of *S. albidoflavus* UO-FLAV-004-FNS1 fed with sakuranetin (green), *S. albidoflavus* UO-FLAV-004 fed with sakuranetin (purple) as control, and genkwanin pure standard (blue). Sakuranetin (SAK); Genkwanin (GNK).

**Additional file 7: Figure S7.** HPLC-DAD chromatograms of *S. albidoflavus* UO-FLAV-004-OsNOMT fed with apigenin (black) and *S. albidoflavus* UO-FLAV-004 fed with apigenin (red) as control. Apigenin (API); Genkwanin (GNK).

**Additional file 8: Figure S8.** HPLC-DAD chromatograms of *S. albidoflavus* UO-FLAV-004-GNK (blue) and co-culture between *S. albidoflavus* UO-FLAV-004-API and *S. albidoflavus* UO-FLAV-004-OsNOMT (red). Sakuranetin (SAK); Genkwanin (GNK).

**Additional file 9: Figure S9.** Generation of the *S. albidoflavus* UO-FLAV-003 strain. A) Agarose gel for PCR verification of the BGC2 deletion event, using the primers “preRHA BGC2 fw” and “UNS8 rev” on the mutant strain *S. albidoflavus* UO-FLAV-003 (lane 1a) and in the parental strain *S. albidoflavus* UO-FLAV-002 (lane 2a), and also using the primers “BGC2 fw” and “BGC2 rev” on the mutant strain *S. albidoflavus* UO-FLAV-003 (lane 1b) and on the parental strain *S. albidoflavus* UO-FLAV-002 (lane 2b). B) Graphical representation of the expected PCR amplifications shown in the agarose gel picture: (a) expected PCR results on the parental and mutant strains using the primers “preRHA BGC2 fw” and “UNS8 rev” (2415 bp); (b) expected PCR results with primers “BGC2 fw” and “BGC2 rev” (2013 bp).

**Additional file 10: Figure S10.** Generation of the *S. albidoflavus* UO-FLAV-004 strain. A) Agarose gel for PCR verification of the BGC5 deletion event, using the primers “BGC5 Recombination checking” and “UNS8 rev” on the mutant strain *S. albidoflavus* UO-FLAV-004 (lane 1a) and in the parental strain *S. albidoflavus* UO-FLAV-003 (lane 2a), and also using the primers “BGC5 Deletion checking FW” and “BGC5 Deletion checking REV” on the mutant strain *S. albidoflavus* UO-FLAV-004 (lane 1b) and on the parental strain *S. albidoflavus* UO-FLAV-003 (lane 2b). B) Graphical representation of the expected PCR amplifications shown in the agarose gel picture: (a) expected PCR results on the parental and mutant strains using the primers “BGC5 Recombination checking” and “UNS8 rev” (2,942 bp); (b) expected PCR results with primers “BGC5 Deletion checking FW” and “BGC5 Deletion checking REV” (1098 bp).

**Additional file 11: Figure S11.**. HPLC-HRESIMS chromatograms of *S. albidoflavus* J1074 (blue) and *S. albidoflavus* UO-FLAV-004 (red). The *m/z* [M-H]^−^ of the final products of paulomycin BGC, paulomenol A and B, 660.2514 and 646.2359, respectively, are extracted in the chromatogram. The chemical structure is shown over the name of each compound.

**Additional file 12: Table S1.** Primers used in this study.

## Data Availability

Data and materials can be obtained from the research group upon request. Sequences accession data have been included in the “Materials and Methods” section.
