# Supplementary figures and images for: Metabolic engineering in Streptomyces albidoflavus for the biosynthesis of the methylated flavonoids sakuranetin, acacetin, and genkwanin
Source: Microb Cell Fact. 2023 Nov 14;22:234. doi: 10.1186/s12934-023-02247-3 (PMC10648386; doi:10.1186/s12934-023-02247-3)

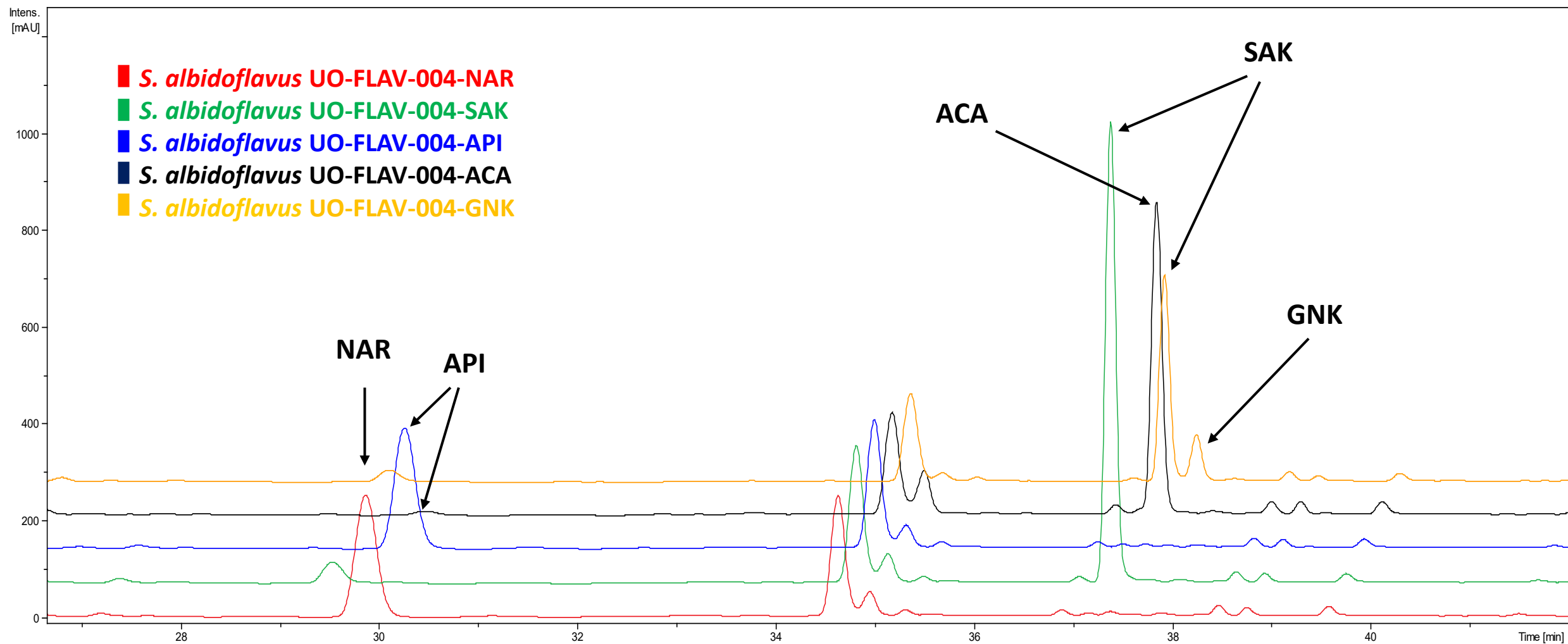

Supplement: Supplementary file 1 — Additional file 1: Figure S1. HPLC-DAD chromatograms of the strains S. albidoflavus UO-FLAV-004-NAR (red), S. albidoflavus UO-FLAV-004-SAK (green), S. albidoflavus UO-FLAV-004-API (blue), S. albidoflavus UO-FLAV-004-ACA (black), S. albidoflavus UO-FLAV-004-GNK (orange). Naringenin (NAR); Sakuranetin (SAK), Apigenin (API); Acacetin (ACA); Genkwanin (GNK). [file 12934_2023_2247_MOESM1_ESM.pdf]

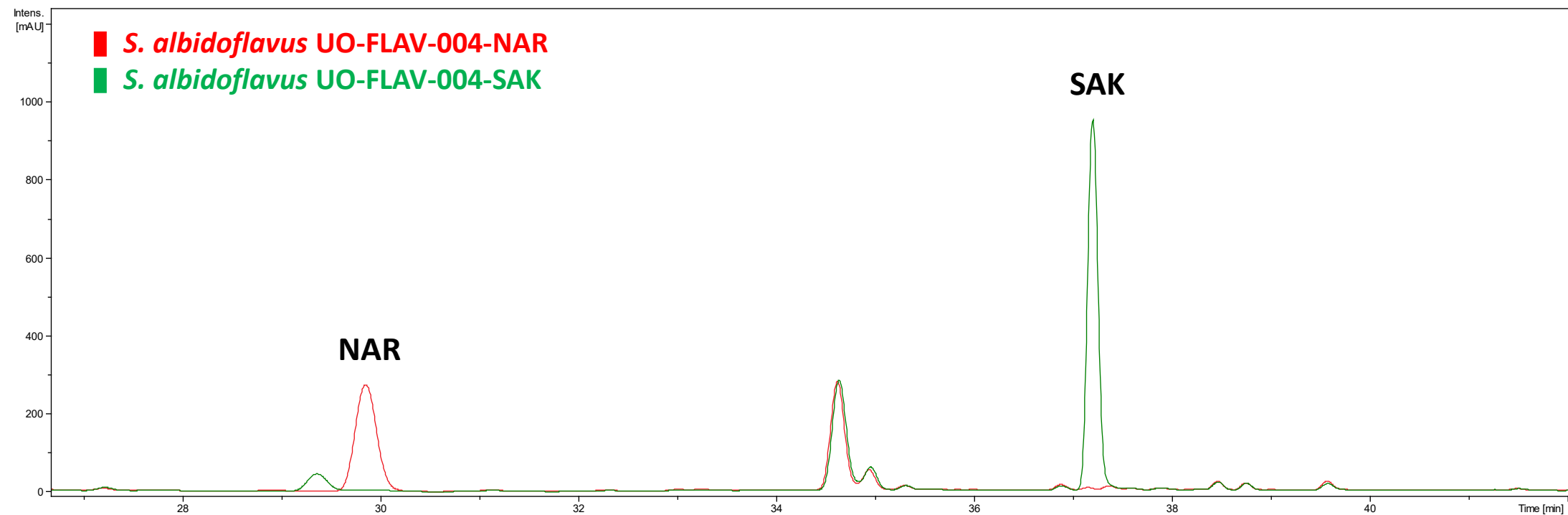

Supplement: Supplementary file 2 — Additional file 2: Figure S2. HPLC-DAD chromatograms of S. albidoflavus UO-FLAV-004-NAR (red) and S. albidoflavus UO-FLAV-004-SAK (green). Naringenin (NAR); Sakuranetin (SAK). [file 12934_2023_2247_MOESM2_ESM.pdf]

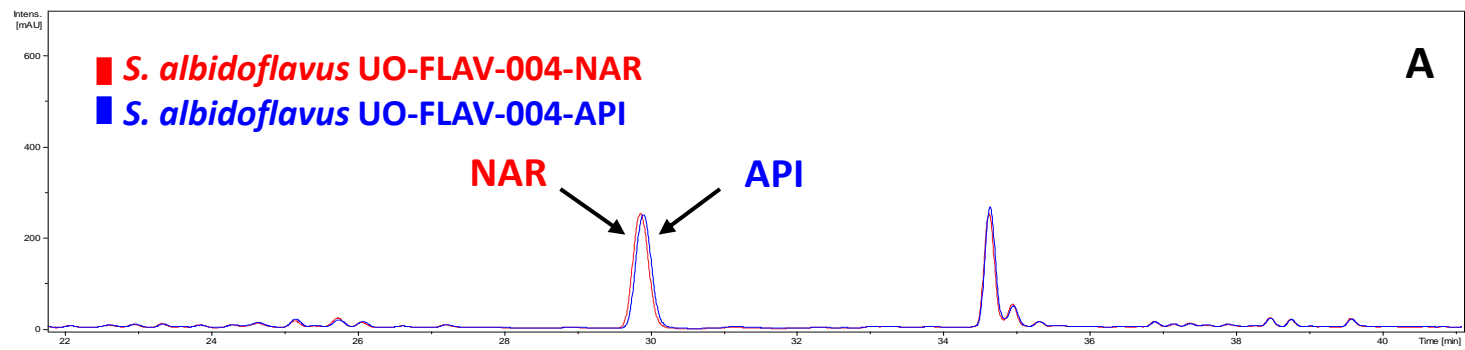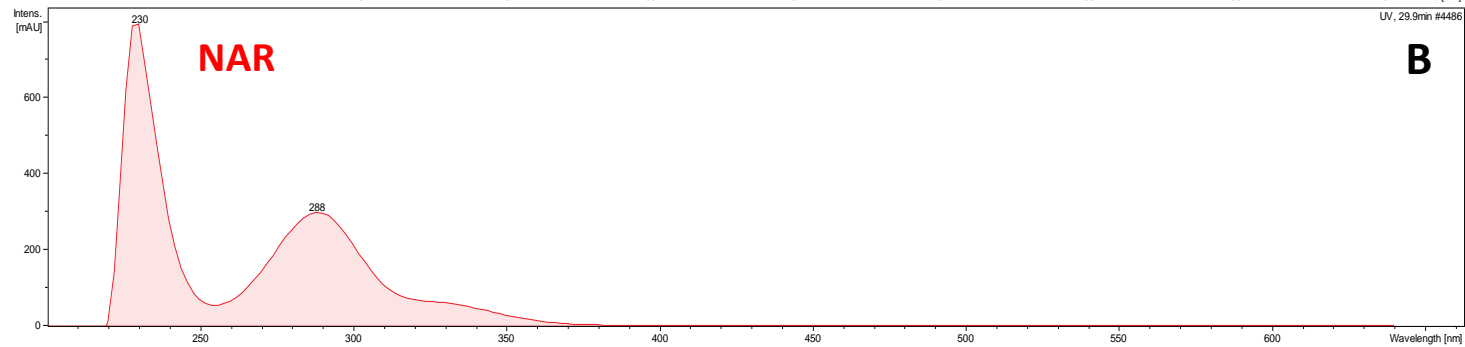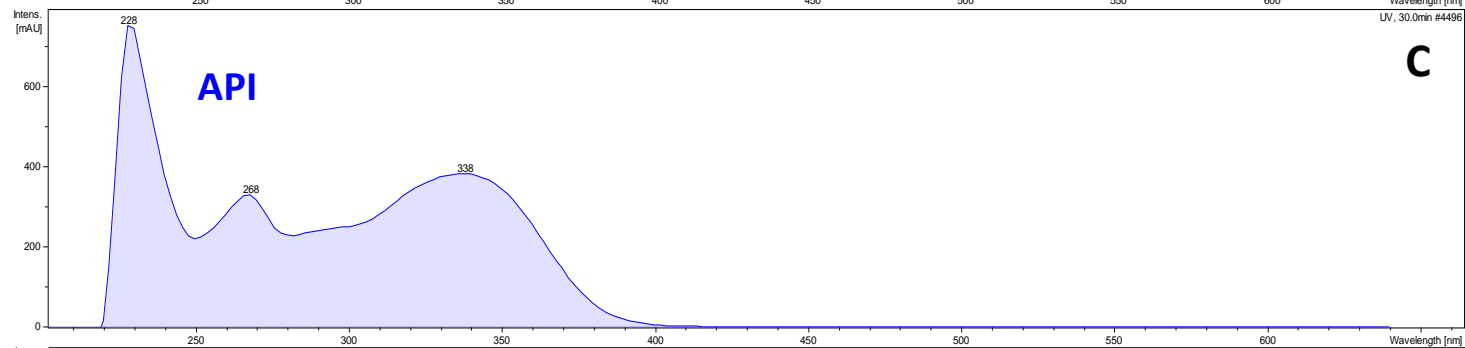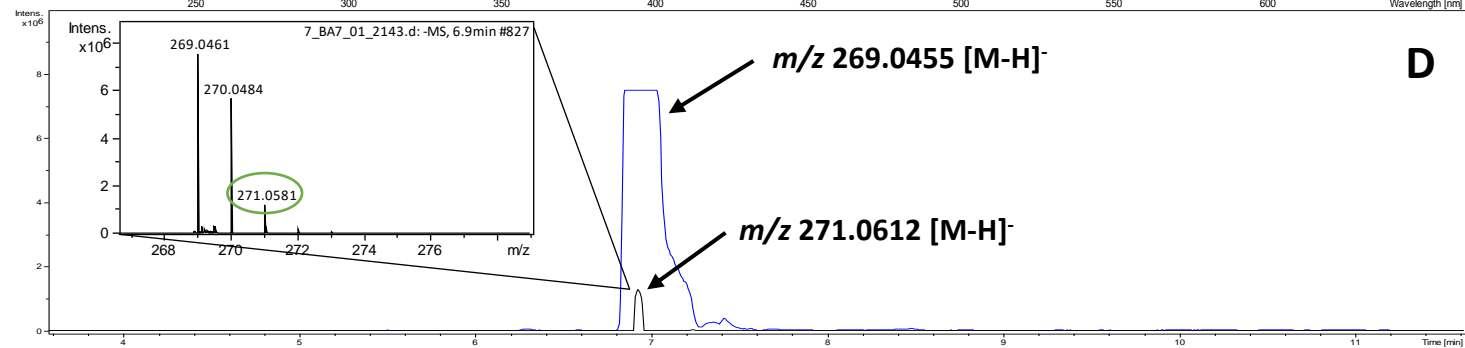

Supplement: Supplementary file 3 — Additional file 3: Figure S3. A) HPLC-DAD chromatograms of S. albidoflavus UO-FLAV-004-NAR (red) and S. albidoflavus UO-FLAV-004-API (blue) showing naringenin and apigenin production, respectively. B) Absorption spectrum of naringenin pure standard in a concentration of 500 µM. C) Absorption spectrum of apigenin pure standard in a concentration of 500 µM. D) Extracted BPCs of apigenin (m/z 269.0455 [M-H]−, blue color) and naringenin (m/z 271.0612 ± [M-H]−, black color) from a sample of the strain S. albidoflavus UO-FLAV-004-API, where only signals from the apigenin isotopic cluster are detected. [file 12934_2023_2247_MOESM3_ESM.pdf]

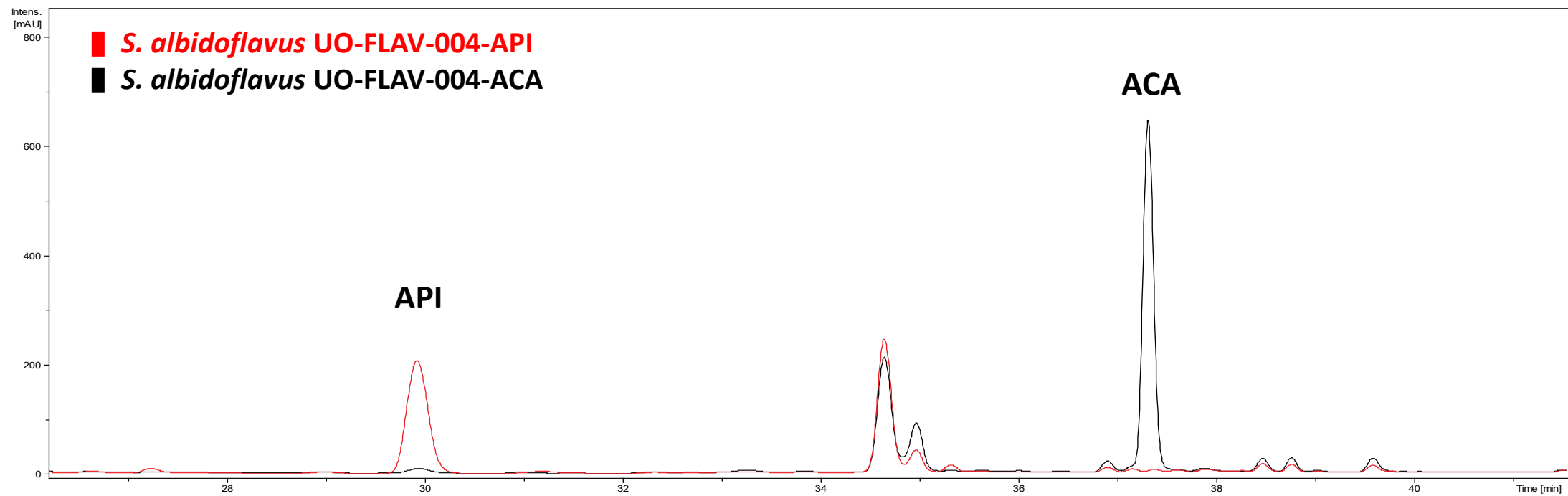

Supplement: Supplementary file 4 — Additional file 4: Figure S4. HPLC-DAD chromatograms of S. albidoflavus UO-FLAV-004-API (red) and S. albidoflavus UO-FLAV-004-ACA (black). Apigenin (API); Acacetin (ACA). [file 12934_2023_2247_MOESM4_ESM.pdf]

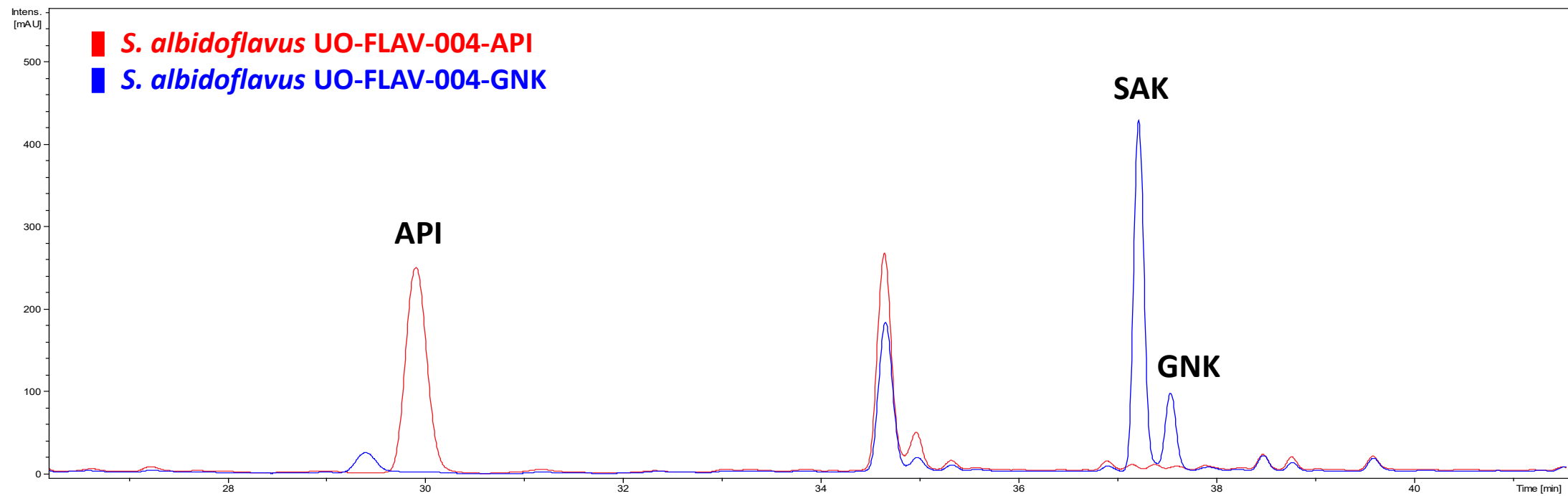

Supplement: Supplementary file 5 — Additional file 5: Figure S5. HPLC-DAD chromatograms of S. albidoflavus UO-FLAV-004-API (red) and S. albidoflavus UO-FLAV-004 GENK (blue). Apigenin (API); Sakuranetin (SAK); Genkwanin (GNK). [file 12934_2023_2247_MOESM5_ESM.pdf]

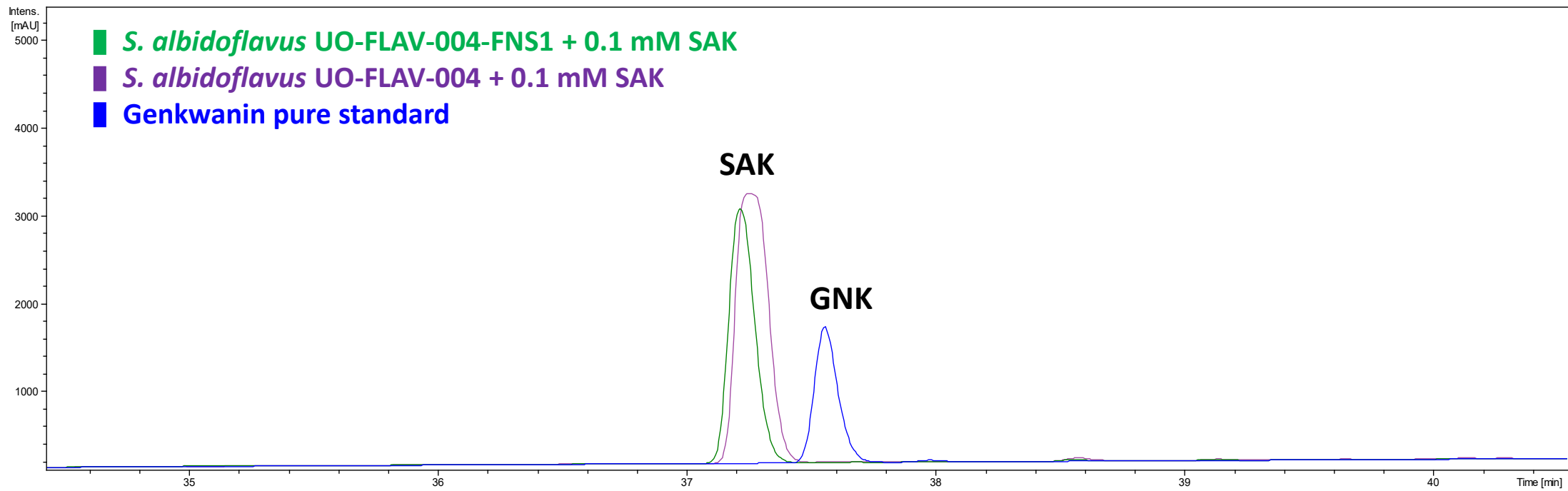

Supplement: Supplementary file 6 — Additional file 6: Figure S6. HPLC-DAD chromatograms of S. albidoflavus UO-FLAV-004-FNS1 fed with sakuranetin (green), S. albidoflavus UO-FLAV-004 fed with sakuranetin (purple) as control, and genkwanin pure standard (blue). Sakuranetin (SAK); Genkwanin (GNK). [file 12934_2023_2247_MOESM6_ESM.pdf]

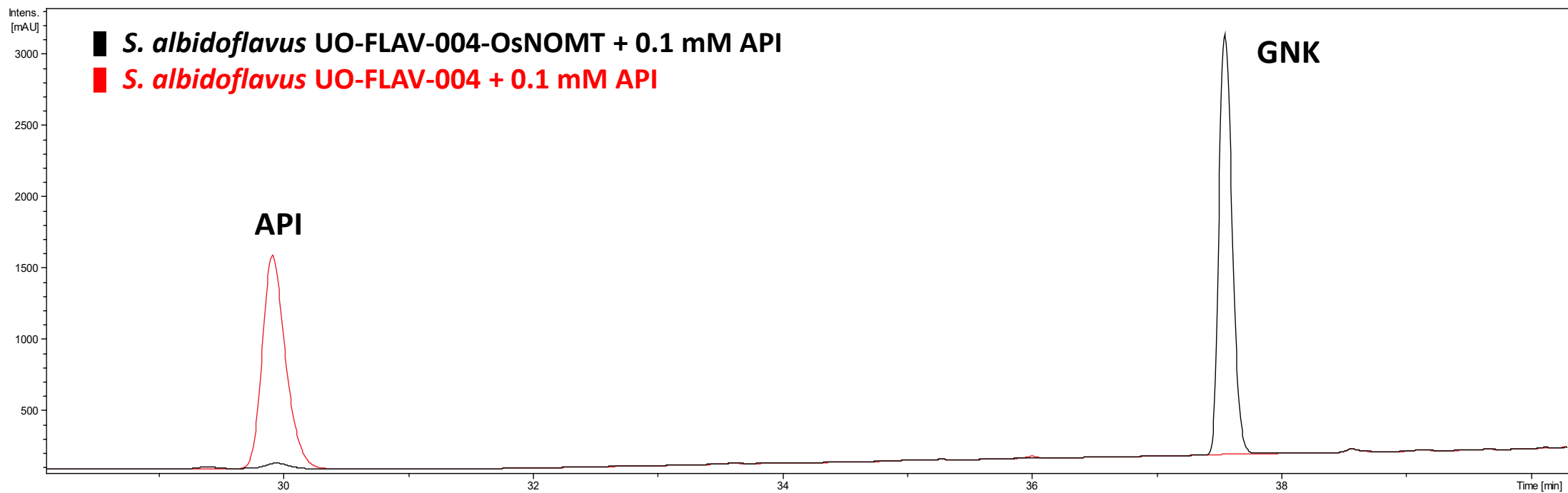

Supplement: Supplementary file 7 — Additional file 7: Figure S7. HPLC-DAD chromatograms of S. albidoflavus UO-FLAV-004-OsNOMT fed with apigenin (black) and S. albidoflavus UO-FLAV-004 fed with apigenin (red) as control. Apigenin (API); Genkwanin (GNK). [file 12934_2023_2247_MOESM7_ESM.pdf]

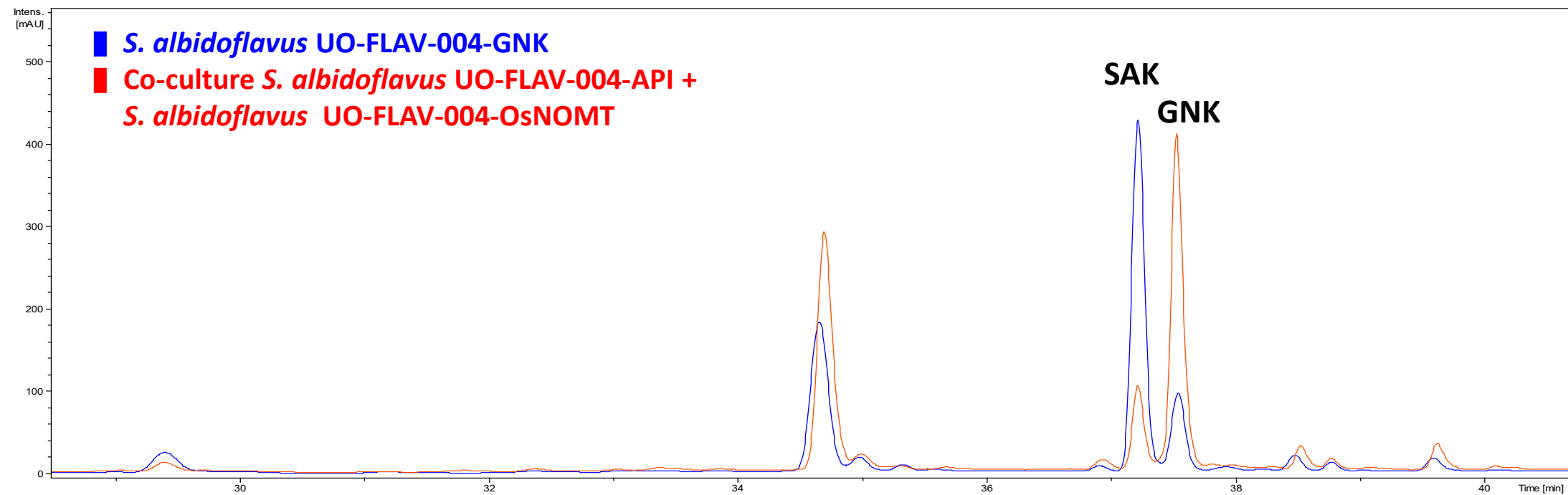

Supplement: Supplementary file 8 — Additional file 8: Figure S8. HPLC-DAD chromatograms of S. albidoflavus UO-FLAV-004-GNK (blue) and co-culture between S. albidoflavus UO-FLAV-004-API and S. albidoflavus UO-FLAV-004-OsNOMT (red). Sakuranetin (SAK); Genkwanin (GNK). [file 12934_2023_2247_MOESM8_ESM.pdf]

**A**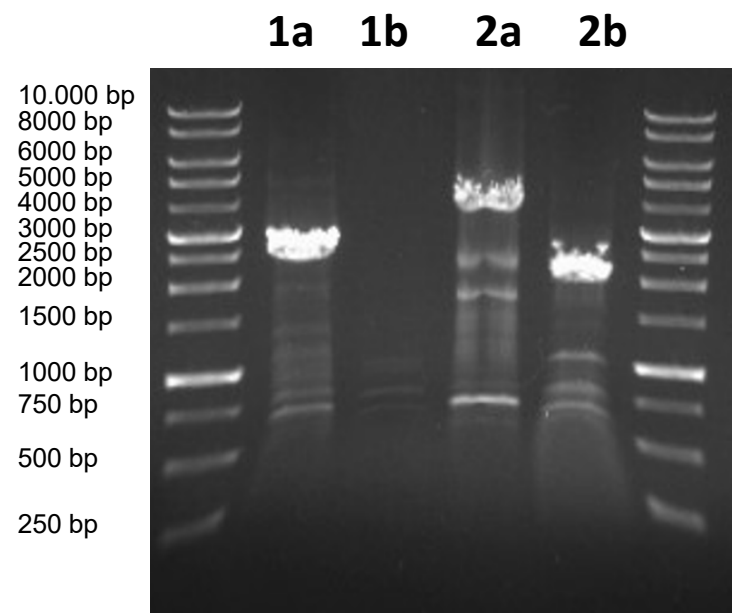**B**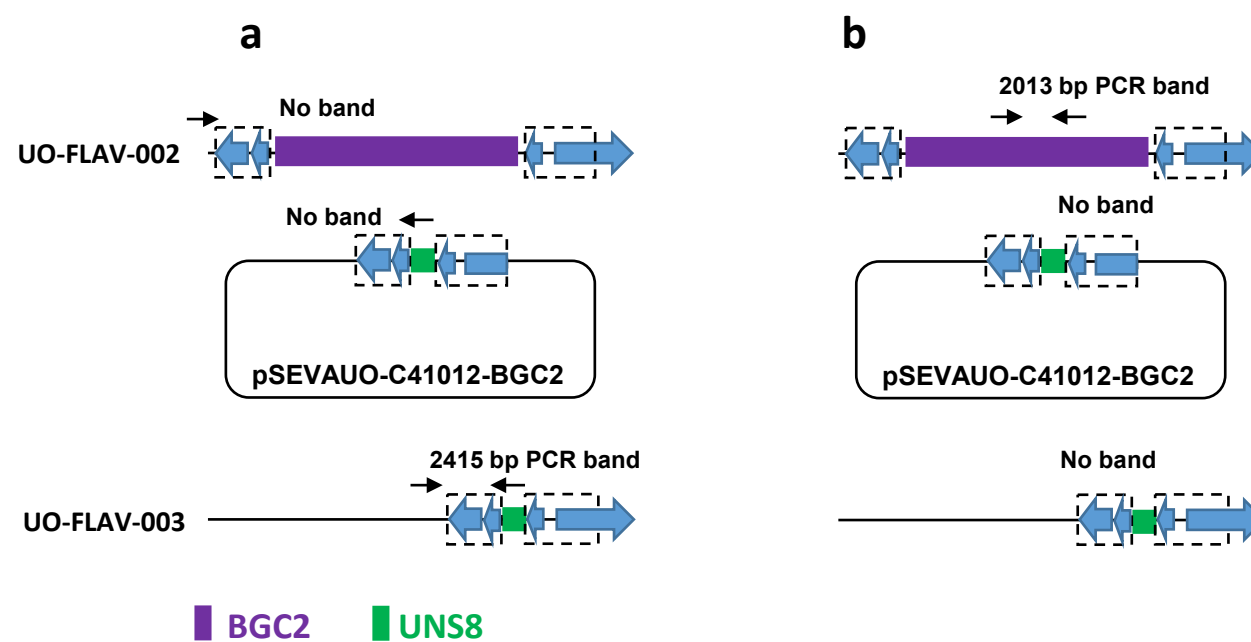

Supplement: Supplementary file 9 — Additional file 9: Figure S9. Generation of the S. albidoflavus UO-FLAV-003 strain. A) Agarose gel for PCR verification of the BGC2 deletion event, using the primers “preRHA BGC2 fw” and “UNS8 rev” on the mutant strain S. albidoflavus UO-FLAV-003 (lane 1a) and in the parental strain S. albidoflavus UO-FLAV-002 (lane 2a), and also using the primers “BGC2 fw” and “BGC2 rev” on the mutant strain S. albidoflavus UO-FLAV-003 (lane 1b) and on the parental strain S. albidoflavus UO-FLAV-002 (lane 2b). B) Graphical representation of the expected PCR amplifications shown in the agarose gel picture: (a) expected PCR results on the parental and mutant strains using the primers “preRHA BGC2 fw” and “UNS8 rev” (2415 bp); (b) expected PCR results with primers “BGC2 fw” and “BGC2 rev” (2013 bp). [file 12934_2023_2247_MOESM9_ESM.pdf]

**A**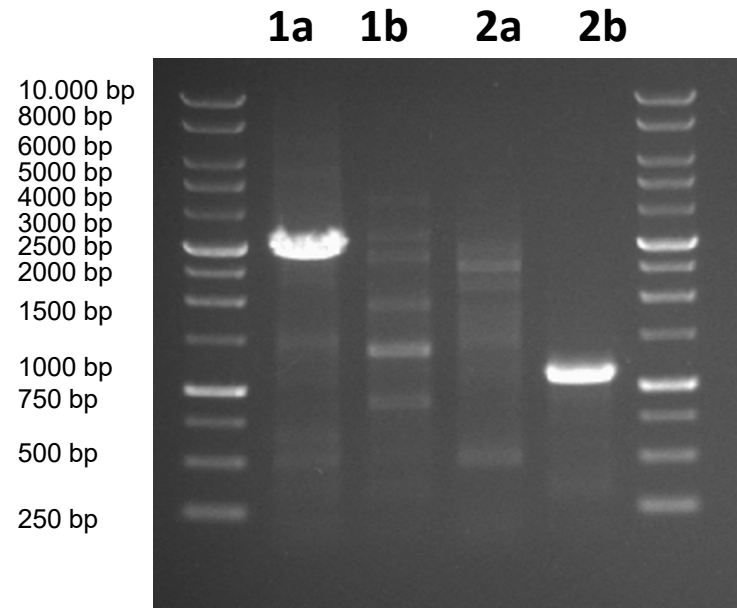**B**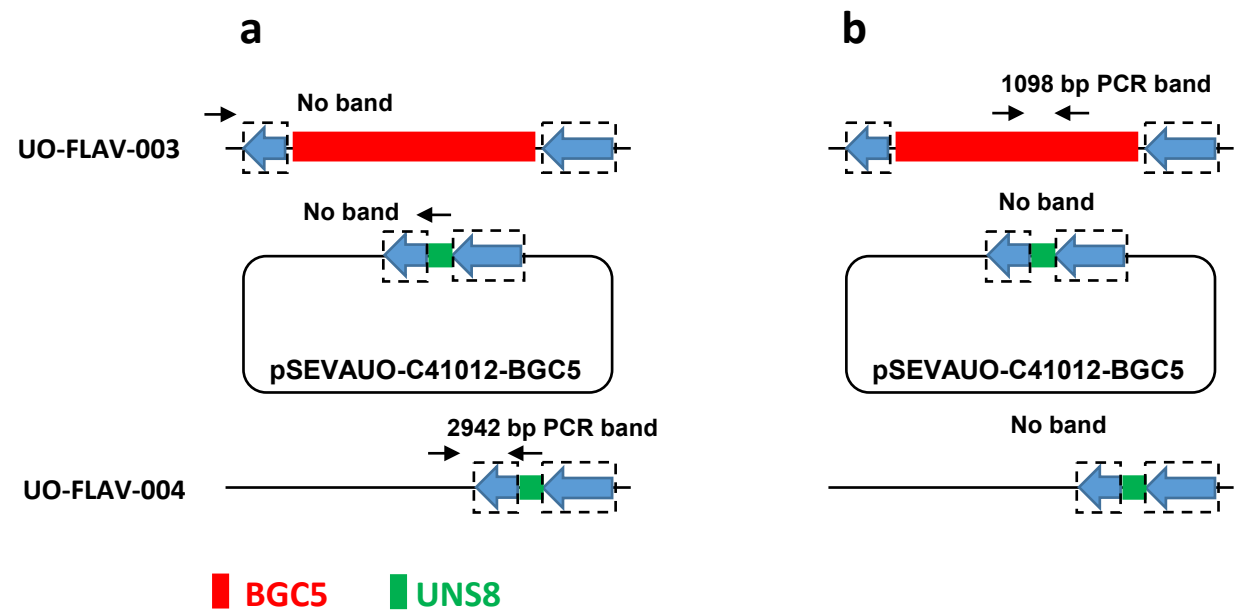

Supplement: Supplementary file 10 — Additional file 10: Figure S10. Generation of the S. albidoflavus UO-FLAV-004 strain. A) Agarose gel for PCR verification of the BGC5 deletion event, using the primers “BGC5 Recombination checking” and “UNS8 rev” on the mutant strain S. albidoflavus UO-FLAV-004 (lane 1a) and in the parental strain S. albidoflavus UO-FLAV-003 (lane 2a), and also using the primers “BGC5 Deletion checking FW” and “BGC5 Deletion checking REV” on the mutant strain S. albidoflavus UO-FLAV-004 (lane 1b) and on the parental strain S. albidoflavus UO-FLAV-003 (lane 2b). B) Graphical representation of the expected PCR amplifications shown in the agarose gel picture: (a) expected PCR results on the parental and mutant strains using the primers “BGC5 Recombination checking” and “UNS8 rev” (2,942 bp); (b) expected PCR results with primers “BGC5 Deletion checking FW” and “BGC5 Deletion checking REV” (1098 bp). [file 12934_2023_2247_MOESM10_ESM.pdf]

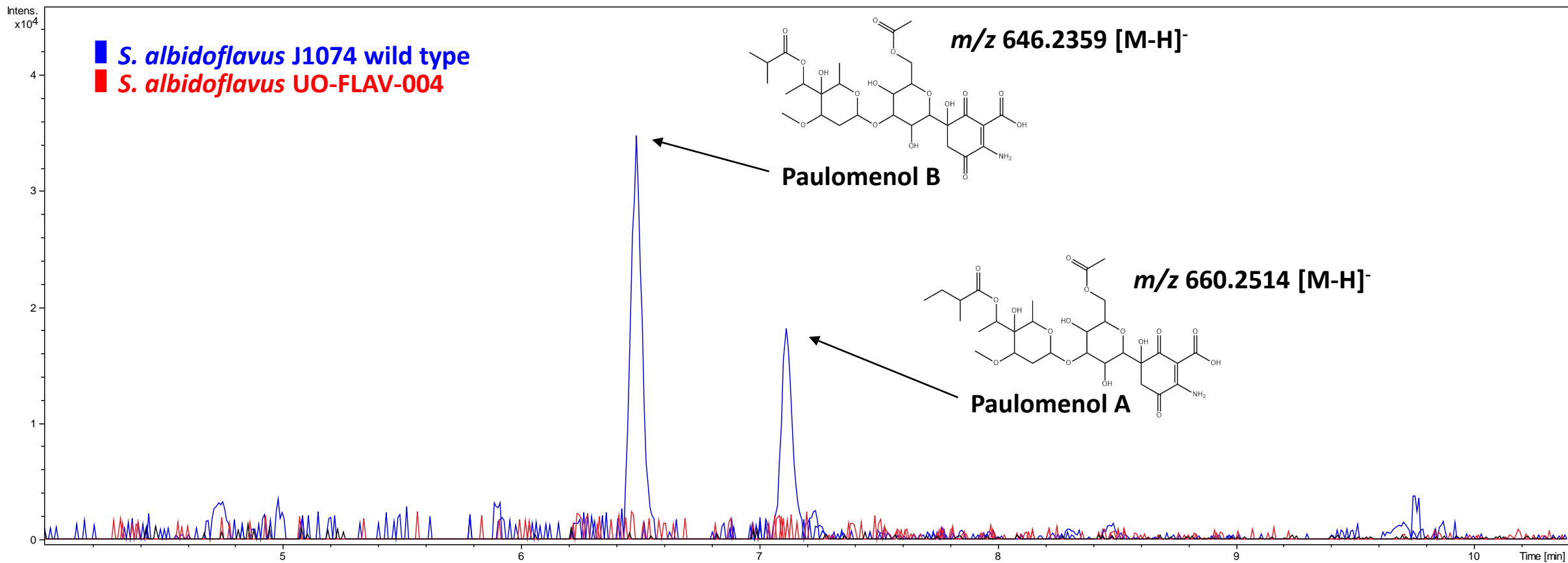

Supplement: Supplementary file 11 — Additional file 11: Figure S11.. HPLC-HRESIMS chromatograms of S. albidoflavus J1074 (blue) and S. albidoflavus UO-FLAV-004 (red). The m/z [M-H]− of the final products of paulomycin BGC, paulomenol A and B, 660.2514 and 646.2359, respectively, are extracted in the chromatogram. The chemical structure is shown over the name of each compound. [file 12934_2023_2247_MOESM11_ESM.pdf]
