# Supplementary material for: Metabolic engineering in Streptomyces albidoflavus for the biosynthesis of the methylated flavonoids sakuranetin, acacetin, and genkwanin
Source: Microb Cell Fact. 2023 Nov 14;22:234. doi: 10.1186/s12934-023-02247-3 (PMC10648386; doi:10.1186/s12934-023-02247-3)
Supplement: Supplementary file 12 — Additional file 12: Table S1. Primers used in this study. [file 12934_2023_2247_MOESM12_ESM.docx]

**Metabolic engineering and culture medium optimization in *Streptomyces albidoflavus* for *de novo* biosynthesis of the methylated flavonoids sakuranetin, acacetin, and genkwanin**

Álvaro Pérez-Valero^1,2,3^, Suhui Ye^1,2,3^, Patricia Magadán-Corpas^1,2,3^, Claudio J. Villar^1,2,3^, Felipe Lombó^1,2,3^*

^1^Research Group BIONUC (Biotechnology of Nutraceuticals and Bioactive Compounds), Departamento de Biología Funcional, Área de Microbiología, Universidad de Oviedo, Oviedo, Principality of Asturias, Spain.

^2^IUOPA (Instituto Universitario de Oncología del Principado de Asturias), Principality of Asturias, Spain.

^3^ISPA (Instituto de Investigación Sanitaria del Principado de Asturias), Principality of Asturias, Spain.

[*corresponding author: lombofelipe@uniovi.es](mailto:*corresponding%20author:%20lombofelipe@uniovi.es)

**Table S1**. Primers used in this study.

| **Function** | **Name** | **Sequence 5’- 3’** |
| --- | --- | --- |
| Homology region A BGC2 | UNS8 Primer1 BGC2 rev | CCAGGTGGTTGATGGGTTGATTGCTTTGGTTGAGACGAGGTTCGGCGGTGCTGGGATG |
|  | UNS7 Primer 2 BGC2 fw | CAAGACGCTGGCTCTGACATTTCCGCTACTGAACTACTCGCTTGAATCGCCTGAGCCCC |
| Homology region B BGC2 | UNS8 Primer 3 BGC2 fw | CCTCGTCTCAACCAAAGCAATCAACCCATCAACCACCTGGCCTCTTCACCCCGCTGCC |
|  | UNS6 Primer4 BGC2 rev | TATGTGACCGTAGAGTATTCTTAGGTGGCAGCGAACGAGGGTCCGCCTTGTGGTGGG |
| Recombination checking BGC2 | preRHA BGC2 fw | gccactggtgtcgctctc |
|  | UNS8 rev | CCAGGTGGTTGATGGGTTG |
| Deletion checking BGC2 | BGC2 fw | CAAGCACCTGAGGGACGAAC |
|  | BGC2 rev | TCGAGGAGGTGGACGTAGTG |
| Protospacer BGC 2 | Target BGC21 sen | ACGCGCGCGTAGCAGCCGGTGGAG |
|  | Target BGC21 antisen | AAACCTCCACCGGCTGCTACGCGC |
| Homology región A BGC5 | Primer A BGC5 FW | CAAGACGCTGGCTCTGACATTTCCGCTACTGAACTACTCGAGGATTACCCAGGCGGCG |
|  | Primer A BGC5 REV | CCAGGTGGTTGATGGGTTGATTGCTTTGGTTGAGACGAGGCGCACACGAAACGGAGCC |
| Homology región B BGC5 | Primer B BGC5 FW | CCTCGTCTCAACCAAAGCAATCAACCCATCAACCACCTGGACGCCGTCTCTCCGCTGG |
|  | Primer B BGC5 REV | TATGTGACCGTAGAGTATTCTTAGGTGGCAGCGAACGAGCTTCCTCCCCGAGACCGC |
| Recombination checking BGC5 | BGC5 Recombination checking | GATCAACCTCGGCATGGACC |
|  | UNS8 REV | CCAGGTGGTTGATGGGTTG |
| Deletion checking BGC5 | BGC5 Deletion checking FW | CAGAAGCCGCAAGTAGGTCA |
|  | BGC5 Deletion checking REV | GTGGTTCAAACGCATGGTCC |
| pSEVA88c1 amplification | Vector FW UNS6 | CTCGTTCGCTGCCACCTAAGAATACTCTACGGTCACATACAAGCTTGCGGCCGCGTCG |
|  | Vector REV UNS7 | CGAGTAGTTCAGTAGCGGAAATGTCAGAGCCAGCGTCTTGCCTAGGCGGCCTCCTGTG |
| Protospacer BGC5 | Prot BGC5 FW | ACGCGGTCAACAGATGATTCAGAC |
|  | Prot BGC 5 REV | AAACGTCTGAATCATCTGTTGACC |
